# Seasonal Activity of Adult Ticks *Ixodes persulcatus* (Acari, Ixodidae) in the North-West of the Distribution Area

**DOI:** 10.3390/ani13243834

**Published:** 2023-12-13

**Authors:** Sergey V. Bugmyrin, Lyubov A. Bespyatova

**Affiliations:** Institute of Biology, Karelian Research Centre, Russian Academy of Sciences, 185910 Petrozavodsk, Russia; gamasina@mail.ru

**Keywords:** *Ixodes persulcatus*, northern border, seasonal activity, weather

## Abstract

**Simple Summary:**

The taiga tick is a common tick species in northern Europe. These blood-sucking parasites transmit dangerous human pathogens, including the causal agents of tick-borne encephalitis and Lyme borreliosis. Knowledge of their ecology is essential for the development of scientifically robust recommendations about the best ways of protecting humans from tick attacks. In our work, we described the seasonal course of activity of the adult taiga ticks. We found that potential risks to humans in the study area were greatest in the second half of May and early June, when the ticks were particularly numerous and active. By mid-summer, the activity of the ticks had decreased to a minimum. Our results, derived from thirty years of monitoring studies, show that the taiga ticks have become much more numerous in the north of their range and that they now become active earlier. These changes in the activity of the ticks may be associated with climate warming.

**Abstract:**

The taiga tick *Ixodes persulcatus* (Schulze, 1930) (Acari, Ixodidae) is the main vector of the tick-borne encephalitis virus and one of the most widespread species of ixodid ticks in the Palaearctic. In this paper, we present long-term data on the seasonal activity of adult ticks in the north-west of their distribution. The seasonal activity of *Ixodes persulcatus* was studied from 1982 to 1990 and from 2012 to 2023 in the middle taiga subzone of Karelia (N62.0697, E33.961). In the study area, adult ticks *I. persulcatus* demonstrate a pronounced spring–summer activity with a unimodal curve of abundance change. A comparison of the monitoring data from the 1980s and the 2010s showed a significant increase in the abundance of *I. persulcatus* in the study area. A tendency towards an earlier start of the tick activity, as compared to the 1980s, is now being observed.

## 1. Introduction

The taiga tick *Ixodes persulcatus* (Schulze, 1930) (Acari, Ixodidae) is a temporary ectoparasite with a prolonged feeding period and pasture-questing type of attack [1]. *Ixodes persulcatus* has a complex life cycle, which includes the egg, the larva, the nymph, and the adult (male or female). Egg development and moulting of all active phases of the life cycle occur in the forest litter. Unfed ticks crawl to the surface of litter and onto vegetation. As a rule, females can climb along shrubs or blades of grass up to 1 m, while nymphs and larvae can only crawl to much lower heights. An extensive host range of *I. persulcatus* comprises about 200 species of mammals and more than 120 species of birds. Adults feed on large- and medium-sized mammals, both wild (ungulates, carnivores, and hares) and domestic (cattle, dogs, and cats). Larvae and nymphs parasitize medium-sized and small mammals (hares, voles, and insectivores), reptiles, and birds [2].

*Ixodes persulcatus* is the main vector of the tick-borne encephalitis virus and one of the most widespread species of ixodid ticks in the Palaearctic. Its boreal range stretches from Sweden to the Pacific Ocean [2,3,4,5]. The seasonal activity of *I. persulcatus* varies across the distribution area due to the differences in the natural and climatic conditions [6,7,8,9].

The first data on the seasonal dynamics of tick abundance in Karelia were obtained in the 1950s. Adult *I. persulcatus* were shown to be active in spring–summer, with the maximum in late May–early June and a sharp decline in July [10,11]. Until recently, the north-western border of distribution of this species passed across Karelia [12,13]. Recent studies have demonstrated an increasing abundance of *I. persulcatus* and the expansion of its distribution northwards and westwards [14,15,16,17]. This species is now widespread in Karelia, and its importance as a vector of dangerous infections cannot be overestimated. Most ticks removed from humans in medical institutions in the Republic of Karelia are *I. persulcatus* (95% of the total number), with adults predominating and nymphs making up only about 0.4% [18]. Pathogens recorded so far in *I. persulcatus* from Karelia are tick-borne encephalitis virus, Alongshan virus, several representatives of the family Phenuiviridae, *Borrelia afzelii*, *Borrelia garinii*, *Anaplasma phagocytophillum*, *Ehrlichia muris*, *Candidatus* Rickettsia tarasevichiae, and *Candidatus* Lariskella arthropodarum [18,19].

Climate change is one of the factors possibly influencing the species dynamics at the periphery of the distribution. It would be interesting to assess the influence of climate change on the usual course of the seasonal activity of *Ixodes persulcatus*. In this paper, we present data on the seasonal activity of adult *I. persulcatus* in Karelia, Russia.

## 2. Materials and Methods

**Figure 1:**
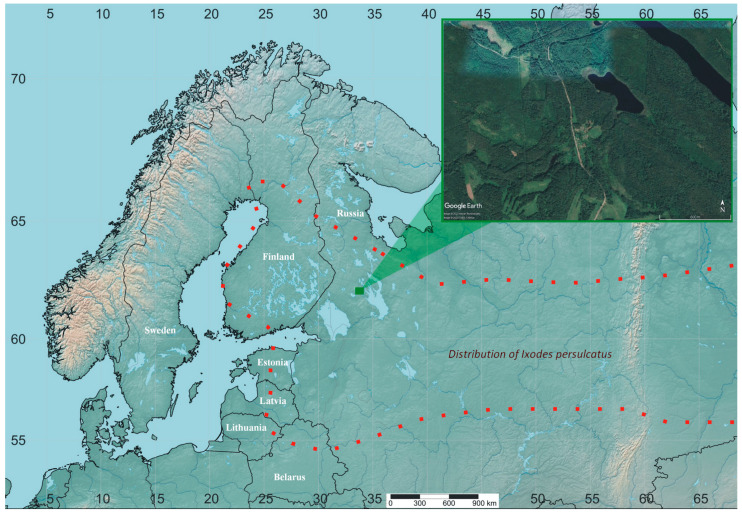
Map of the study area (Gomselga field station, Russia, 62.07° N, 33.96° E). The map was made in SimpleMappr [20].

Seasonal activity of adult ticks *Ixodes persulcatus* was studied from 1982 to 1990 and from 2012 to 2023 in the middle taiga subzone of Karelia (Gomselga field station of the Institute of Biology, Karelian Research Centre, Russian Academy of Sciences, 62.0697° N, 33.961° E) (Figure 1).

Information on the abundance of *I. persulcatus* from 1982 to 1990 was taken from the archives of the Karelian Research Centre of the Russian Academy of Sciences. Tick surveys were carried out from April to July at intervals of about 10 days (by flagging from vegetation). Between three and nine surveys were made each year. The seasonal activity of *I. persulcatus* was not monitored between 1991 and 2011.

From 2012 to 2023, the surveys were conducted in the snowless period from April (immediately after the melting of the snow cover) to November (until the formation of a continuous snow cover). The data were obtained from surveys made every ten days between April and August on a monitoring route in a mixed birch–pine secondary forest [14]. Additionally, one-time control surveys were made in September, October, and November.

Ixodid ticks were collected from vegetation by flagging. The collected ticks (1232 males and 1335 females) were identified according to the available identification keys [2]. We found only four *I. persulcatus* nymphs in the 2012–2023 study period. They were fixed, and their species was later ascertained. We did not estimate the abundance of nymphs, as their ratio in collections from vegetation is very low.

**Figure 2:**
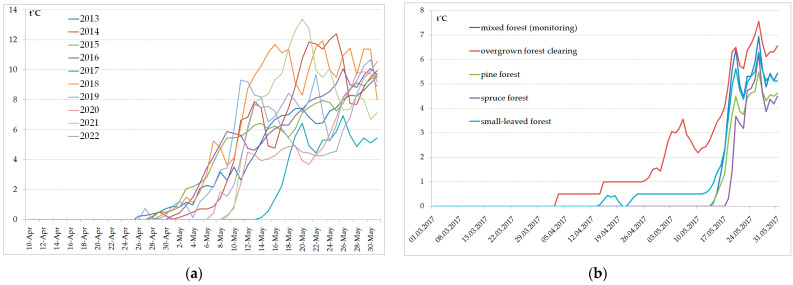
Average daily litter temperature along the monitoring route (**a**) and in five different biotopes (**b**); measurements taken every 3 h.

Air temperature and relative humidity were recorded every 2 h with the help of a Hygrochron DS1923 data logger. The logger was fixed at a height of about 2 m from the ground surface. Thermochron DS1921 data loggers recording only temperature every 3 h were placed in the litter (down to a depth of 5 cm) in five types of biotopes: mixed forest (monitoring); small-leaved forest; pine forest; spruce forest; and overgrown forest clearing. The data array was exported to Access, where queries to calculate average daily air and litter temperatures were created (Figure 2).

## 3. Results

In the study area, adult ticks *I. persulcatus* demonstrated a pronounced spring–summer activity characterised by a unimodal curve of abundance change.

**Figure 3:**
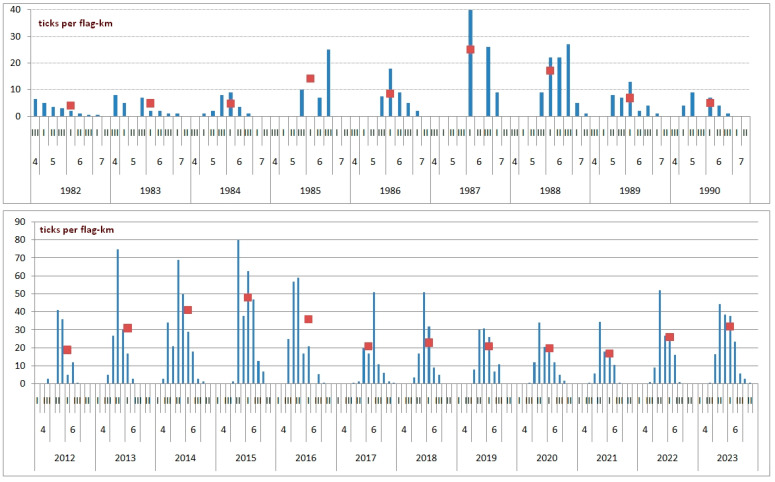
The course of seasonal activity of ticks *Ixodes persulcatus* during two long-term observation period lines (squares—average values).

During the first monitoring period, from 1982 to 1990, seasonal averages of *I. persulcatus* abundance calculated based on 3–5 highest values ranged from 4 (1982) to 25 (1987) ticks per flag-km. Peak abundance was usually recorded in June (in 6 out of 9 years of observations) (Figure 3).

**Figure 4:**
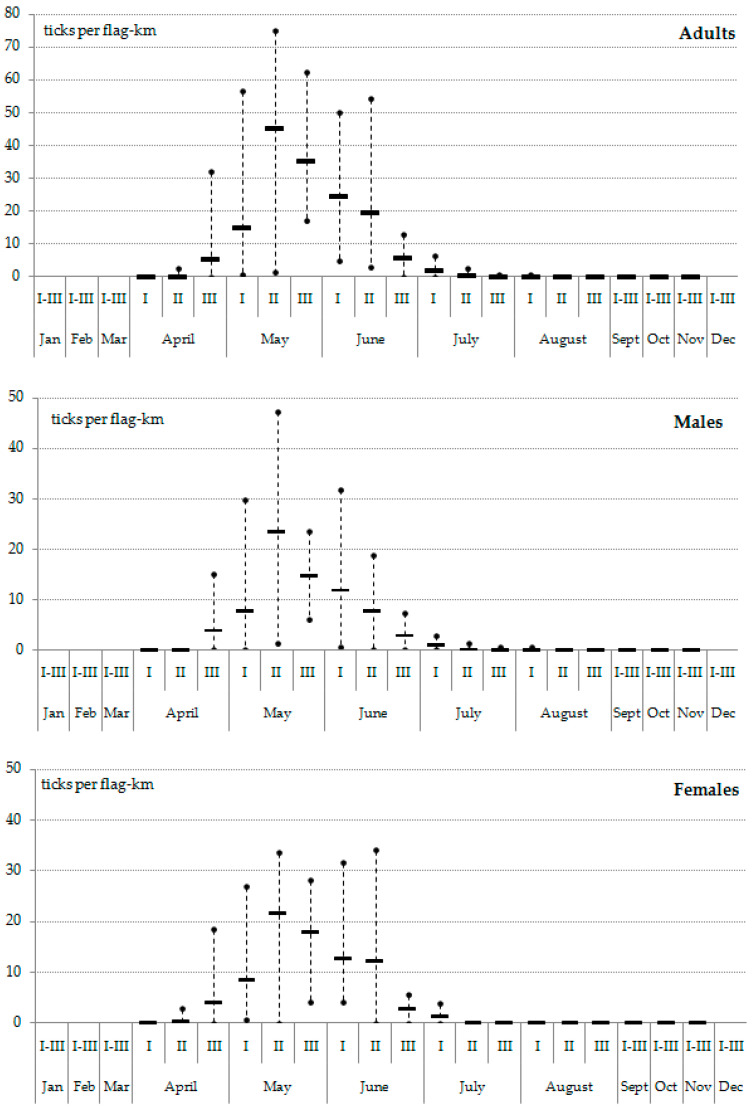
Changes in the abundance of *Ixodes persulcatus* (adults, males, and females) during the activity season in the middle taiga subzone of Karelia. Generalised data for 2012–2023; lines—average values; points—extreme values.

**Figure 5:**
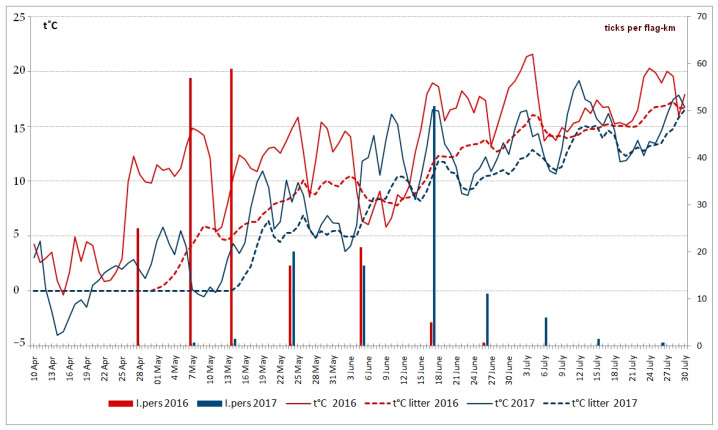
Results of surveys of relative abundance (ticks per flag-km) of adult ticks *Ixodes persulcatus* (I.pers) made during ten-day periods in 2016 and 2017.

In 2012–2023, the relative abundance of the ticks in the monitoring area increased significantly as compared to the first monitoring period. The seasonal average of the five highest values was never less than 18 ticks per flag-km (Figure 3). Adult ticks were active, on average, for 74 days. The shortest activity period (62 days) was recorded in 2018, while the longest period (87 days) was recorded in 2014. The abundance of *I. persulcatus* usually reached the peak in the second ten-day period of May (9 out of 12 years of observations). The long-term average abundance during this time made up about 45 ticks per flag-km (Figure 4). In 2017, the activity of *I. persulcatus* reached the peak in mid-June, and the ticks were observed along the route until the end of July (Figure 5). This is unusual for Karelia. The seasonal activity of males and females of *I. persulcatus* was similar, with the main difference being a sharper decrease in the abundance of males after the peak (Figure 4). Monitoring data (2012–2023) show that the ratio of males and females in the collections generally did not differ (*p* > 0.05) from the theoretically expected one (1:1) in different years. It was different from the theoretically expected one (m:f = 0.6:1; *p* = 0.01) only in the second ten-day period of June (pooled samples from 2012 to 2023).

The date when the average daily temperature in the forest litter first exceeded 0 °C varied in different years in the range of 20 days in one biotope (Figure 2a) and in the range of up to 35 days in different biotopes (Figure 2b). The start of *I. persulcatus* activity was noted at average daily air and litter temperatures of (+3–+8 °C) and (0–+3 °C), respectively. During the activity peak, the average daily temperatures were (+5–+15 °C) and (+4–+11 °C) for air and litter, respectively (Table 1).

## 4. Discussion

Our results obtained in the north-west of the distribution of the taiga tick agree with the established paradigm of its seasonal dynamics across the range [8,21,22,23]. The ticks become active immediately after the melting of the snow cover, and a rapid increase in abundance in May is followed by a sharp decline in mid-summer. This pattern is largely determined by the obligatory behavioural diapause of *I. persulcatus*, which prevents post-nymphal activation of adults in late summer and autumn [24,25]. Ticks starve during the winter and do not become active until after moulting in the following year. In August, only very few individuals of *I. persulcatus* were found in flagging collections from vegetation in other parts of Karelia (southern Karelia, islands in Lake Onega). The latest record of a feeding female of *I. persulcatus* in Karelia was made on 13 September [18].

The bimodal curve of seasonal dynamics is characteristic of a close species, *Ixodes ricinus* [25], but we do not know any examples of the bimodal curve for *I. persulcatus* in other parts of its range. The main differences between the “northern” and “southern” populations of *I. persulcatus* are due to the dates of the start of activity and the duration of the active period [6,7,8]. Alternating increases and decreases in abundance are often observed during the activity season of *I. persulcatus* (e.g., in Karelia in 2014 and 2015). The reason behind these “stochastic” changes is associated with the changeable weather in spring and early summer, when periods with favourable temperatures are followed by sudden colds or frosts, which temporarily lower the activity of the ticks.

Four types of seasonal dynamics are described for adult *I. persulcatus* ticks throughout their distribution area, differing in the duration of the active period [7,9]. In Karelia, the ticks are characterised by the third type, with a relatively short activity season of 65–80 days. In general, these data agree with the results of our monitoring (74 days on average). It has been shown in mark–recapture studies that males and females of *I. persulcatus* are present in the study area for at least 33 days and 51 days, respectively [26]. Data from experiments in cages made in the 1950s indicate that females of *I. persulcatus* are active for 2–2.5 months after overwintering [10].

The exact dates when the taiga ticks become active depend on a complex of abiotic factors: weather conditions in spring; snow cover depth; microclimatic characteristics of the biotope, etc. Continuous recordings of litter temperature in different biotopes have been carried out near the Gomselga field station since 2013. Based on these data, we can conclude that large-scale activation of ixodid ticks was observed in a wide range of average daily temperature values (Table 1). At the same time, the abundance of active ticks varied significantly during the same periods in different years (Figure 4). A comparison of two adjacent years provides a demonstrative example (Figure 5). In 2016, the seasonal activity of *I. persulcatus* was typical for Karelia. The first active ticks were found in the third ten-day period of April. A sudden warming in the second half of April resulted in a rapid melting of the snow cover and in the warming of the litter to optimal temperature, and the abundance of *I. persulcatus* peaked in early May. In 2017, the temperatures in April–May were low, the snow melted late, and the entire course of the seasonal activity of the ticks was significantly altered. The peak of activity was recorded in mid-June, with active ticks being present continuously until the end of July (Figure 5).

The intensity of snow melting and soil warming, which determine the start of tick activity, obviously depends not only on the year of study but also on the characteristics of the biotope. The snow cover melts much earlier in an overgrown forest clearing than in forest habitats. We found that biotopic differences in soil temperature were greater than interannual differences (Figure 2). This means that the total seasonal activity of the ticks is likely to be longer if the landscape in the study area is more diverse.

The first thing that becomes evident after comparing the monitoring data from the 1980s and the 2010s is a significant increase in the abundance of *I. persulcatus* in the study area. It is largely explained by the general climatic trend [14]. At present, a certain tendency towards an earlier start of tick activity, as compared to the 1980s, is observed. This is consistent with the results of long-term phenological observations made in the Kivach Reserve, 20 km north of the study area [27]. The time of emergence of the first ticks is determined by a single factor, the melting of the snow cover [28], and its long-term trend is a shift to earlier dates [29]. An earlier activation of *I. persulcatus* in the northern part of the distribution should contribute to the successful implementation of the life cycle (feeding and subsequent development) of greater numbers of ticks.

## 5. Conclusions

Our results obtained in the north-west of the distribution of the taiga tick (*Ixodes persulcatus*) agree with the established paradigm of its seasonal dynamics across the range. The ticks become active immediately after the melting of the snow cover, and a rapid increase in abundance in May is followed by a sharp decline in mid-summer. In the study area, adult ticks were active, on average, for 74 days. In 2012–2023, the relative abundance of ticks in the monitoring area increased significantly as compared to the first monitoring period (1980s). A tendency towards an earlier start of the tick activity, as compared to the 1980s, is now being observed. This shift may be associated with climate warming.

## Figures and Tables

**Table 1:** Average daily litter and air temperatures along the monitoring route in the beginning and at the peak of seasonal activity of *Ixodes persulcatus*.

Year	Beginning of Activity			Peak of Activity		
	Ten-Day Period/Month	Temperature, °C		Ten-Day Period/Month	Temperature, °C	
		Litter	Air		Litter	Air
2013	III/04	2	6	II/05	6	13
2014	II/04	0	5	II/05	7	12
2015	I/05	3	8	II/05	6	9
2016	III/04	0	6	II/05	6	10
2017	I/05	0	3	II/06	10	13
2018	I/05	3	8	III/05	11	14
2019	I/05	2	7 *	III/05	8	10
2020	III/04	0	2 *	II/05	4	5 *
2021	III/04	0	3 *	II/05	7	15 *
2022	III/04	0	3 *	II/05	6	8 *
2023	III/04	2	7 *	II/05	10	12 *

* data obtained at the meteorological station situated 20 km to the north-east of the study area (WMO ID 22727).

## Data Availability

The data presented in this study are available on request from the corresponding author. The data are not publicly available due to privacy or ethical restrictions.
